# Plasmonics and its Applications

**DOI:** 10.3390/ma12091502

**Published:** 2019-05-08

**Authors:** Grégory Barbillon

**Affiliations:** EPF-Ecole d’Ingénieurs, 3 bis rue Lakanal, 92330 Sceaux, France; gregory.barbillon@epf.fr

**Keywords:** plasmonics, sensing, surface-enhanced Raman scattering, sum-frequency generation, third harmonic generation, surface-enhanced fluorescence, metasurfaces, catalysis, lanthanum hexaboride, solar cell

## Abstract

Plasmonics is a quickly developing subject that combines fundamental research and applications ranging from areas such as physics to engineering, chemistry, biology, medicine, food sciences, and the environmental sciences. Plasmonics appeared in the 1950s with the discovery of surface plasmon polaritons. Then, plasmonics went through a novel impulsion in mid-1970s when the surface-enhanced Raman scattering was discovered. Nevertheless, it is in this last decade that a very significant explosion of plasmonics and its applications has occurred. Thus, this special issue reports a snapshot of current advances in these various areas of plasmonics and its applications presented in the format of several articles and reviews written by worldwide researchers of this topic.

## 1. Introduction

Plasmonics (or nanoplasmonics) is a young topic of research, which is part of nanophotonics and nano-optics. Plasmonics concerns to the investigation of electron oscillations in metallic nanostructures and nanoparticles (NPs). Surface plasmons have optical properties, which are very interesting. For instance, surface plasmons have the unique capacity to confine light at the nanoscale [1,2,3]. Moreover, surface plasmons are very sensitive to the surrounding medium and the properties of the materials on which they propagate. In addition to the above, the surface plasmon resonances can be controlled by adjusting the size, shape, periodicity, and materials nature. Indeed, the technological progress allows researchers to produce new plasmonic systems by controlling all the parameters described previously [4,5,6,7,8,9,10,11,12,13,14]. Moreover, theoretical, computational, and numerical simulation tools have been developed in this last decade, allowing for a better understanding of the optical properties of plasmonic systems [1]. Thus, all these optical properties of plasmonic systems can enable a great number of applications, such as biosensors [15,16,17,18,19,20], optical devices [21,22,23,24], and photovoltaic devices [25,26,27,28].

## 2. Synopsis

This special issue is composed of five review articles, five research articles, and two communications. The first part of the latter is devoted to the applications of plasmonics to physics and engineering [29,30,31,32,33]. Concerning the applications to physics, such as non-linear optics, Mattox et al. demonstrated the control of plasmonic properties of LaB${}_{6}$ via Eu-doping on a spectral range from near-infrared to infrared [29]. Then, Mattox et al. presented a review on the tuning of the plasmonic resonance of lanthanum hexaboride for a potential application to solar heat absorption [30]. Besides, Ogata et al. investigated the effect of the plasmonic resonance of metallic nanostructures on the optical third harmonic generation (THG) enhancement of nickel nanostructure-covered microcubes [31]. For the application to photovoltaics, Hajjiah et al. presented a simulation study of the efficiency enhancement of peroskite solar cells by using plasmonic nanoparticles [32]. To finish this first part dedicated to physics with the application to metasurfaces, Li et al. proposed a novel computational method in order to optimize the coupling of the electric fields of a metasurface consisting of nanorod plasmonic antennas. This novel computational method is based on the coupling of the decomposition into several orders [33].

In the second and last part, the discussed topics are devoted to chemistry and sensing, such as surface-enhanced fluorescence, surface-enhanced Raman scattering (SERS), sum-frequency generation (SFG) spectroscopy, and electrocatalysis by using plasmonics [34,35,36,37,38,39,40]. Concerning the surface-enhanced fluorescence, Lu et al. numerically demonstrated a high enhancement effect of the fluorescence signal obtained with a hybrid metal-dielectric nano-aperture antenna consisting of silicon and gold layers [34]. Besides, for the SERS topic, Magno et al. showed excellent analytical enhancement factors of the SERS signal obtained with hybrid Al/Si nanopillars for the detection of thiophenol molecules. These hybrid Al/Si nanopillars have been realized with a simple and quick fabrication technique [35]. Moreover, Sarychev et al. presented a review on the light concentration by metal-dielectric micro/nano-resonators for efficient SERS sensing. In this review, the recent advances in this topic of metal-dielectric micro/nano-resonators for SERS are exposed [36]. Furthermore, D’Orlando et al. showed the feasibility to carry out and control nanostructures of gold nanoparticles, which can be seen as plasmonic molecules whose optical resonances are tuned by modifying the shape, symmetry, and interparticle distances with an AFM (Atomic Force Microscope) device coupled with an optical spectrometer [37]. To complete the sensing part, Han et al. presented a short review on plasmonic biosensing based on the design of nanovoids in thin films by reviewing resonance modes, materials, and hybrid functions using simultaneously electrical conductivity [38]. In addition, Humbert et al. presented a review on the sum-frequency generation (SFG) spectroscopy of plasmonic nanomaterials. In this review, the authors introduced the fundamentals of SFG spectroscopy. Then, they presented an overview of studies of plasmonic nanomaterials by this SFG spectroscopy over the last five years [39]. To conclude this part, as well as this special issue dedicated to “*Plasmonics and its Applications*”, Subramanian et al. presented a review on the electrocatalysis induced by plasmon with multi-component nanostructures. Indeed, the authors highlight the recent progress obtained in the synthesis of these multi-component nanostructures, especially for the plasmonic electrocatalysis of major fuel-forming and fuel cell reactions [40].

## 3. Conclusions

In making this special issue on plasmonics and its applications, I had the pleasure of obtaining contributions from high-quality authors worldwide, and I thank them for that. To conclude, I hope that this special issue dedicated to plasmonics and its applications will be read with interest by the students or researchers who wish to be involved in this topic or to gain an advanced understanding of it.
